# Paired Housing or a Socially-Paired Context Decreases Ethanol Conditioned Place Preference in Male Rats

**DOI:** 10.3390/brainsci12111485

**Published:** 2022-11-02

**Authors:** Eva Lorenz, Chase Moye, Kah-Chung Leong

**Affiliations:** Department of Psychology, Trinity University, San Antonio, TX 78212, USA

**Keywords:** ethanol, social interaction, conditioned place preference, alcohol use disorder

## Abstract

Alcohol abuse dramatically affects individuals’ lives nationwide. The 2020 National Survey on Drug Use and Health (NSDUH) estimated that 10.2% of Americans suffer from alcohol use disorder. Although social support has been shown to aid in general addiction prevention and rehabilitation, the benefits of social support are not entirely understood. The present study sought to compare the benefits of social interaction on the conditioned ethanol approach behavior in rats through a conditioned place preference (CPP) paradigm in which a drug is paired with one of two distinct contexts. In experiment 1A, rats were single-housed and received conditioning trials in which ethanol was paired with the less preferred context. In experiment 1B, rats underwent procedures identical to experiment 1A, but were pair-housed throughout the paradigm. In experiment 1C, rats were single-housed, but concurrently conditioned to a socially-paired context and an ethanol-paired context. By comparing the time spent between the ethanol-paired environment and the saline-paired or socially-paired environment, we extrapolated the extent of ethanol approach behavior in the pair-housed, single-housed, and concurrently conditioned rats. Our results revealed that social interaction, both in pair-housed animals or concurrently socially-conditioned animals, diminished the ethanol approach behavior, which highlights the importance of social support in addiction prevention, treatment, and recovery programs.

## 1. Introduction

The 2020 National Survey on Drug Use and Health (NSDUH) [1] estimated that 10.2% of Americans suffer from alcohol use disorder (AUD). The CDC estimated that alcohol-induced deaths increased from 30,722 in 2014 to 49,061 in 2020 [2]. The negative effects of alcohol use have been consistently demonstrated but have nonetheless increasingly proliferated within the United States. Though there are few consistently effective treatments and interventions for AUD, research suggests that the presence of a strong social support system leads to many positive outcomes for alcohol dependence. In individuals undergoing treatment for alcoholism, household support is strongly associated with less severe alcohol use, while community support significantly correlates with less severe outcomes of alcohol use (i.e., employment problems and legal issues) [3]. Alternatively, social isolation has been shown to increase the likelihood to binge drink alcohol [4], while also increasing likelihood to relapse into alcohol abuse patterns after a period of abstinence [5]. These preliminary reports indicate that social interaction and support may offer an effective intervention to diminish the development and relapse of alcohol dependence.

Behavioral rodent models of social interaction can often be categorized into two primary conditions of conspecific exposure: either through permanent social housing conditions or periods of temporary/acute social interaction. Social housing conditions consist of housing an animal with two or more conspecifics within their home cage [6,7], whereas acute periods of social interaction include consistent but temporary exposure to a conspecific [8,9,10]. While these distinct kinds of social interaction models have sparsely been used to study alcohol dependence, both have been employed to examine the social interactions effect on similar rodent behaviors.

Both social housing and acute social interaction paradigms have successfully modulated changes in reward-related behaviors and stress/anxiety behaviors. For example, rodent studies employ a group-housing or paired-housing model to demonstrate the benefits of social interaction on decreasing the intake of many drugs of abuse. When housed with a same sex partner, female rats self-administered cocaine and methamphetamine at greatly diminished rates compared to female rats housed in isolation [11,12]. Similarly, rats living in pairs consumed much less morphine solution than those in isolation [13]. Studies employing an acute social interaction model have demonstrated similarly diminished reward-related behavior. Social-reward conditioned place preference (CPP) offers another model of acute social interaction through conspecific-paired contextual conditioning. These paradigms have revealed the rewarding potential of social interaction [9]. When presented with the choice between a social-interaction paired context or a cocaine paired context, rats have been shown to prefer the social-interaction paired context [14]. The social interaction paired context was similarly preferred over an amphetamine paired context [15]. Although various rodent models have been used to investigate social interaction’s effect on drug-seeking behavior, few studies have investigated either paradigm’s effect on alcohol-reward behaviors, specifically.

The goal of the present study was to determine whether social interaction, either through a social housing paradigm or concurrent social reward-paired context, reduces reward-related behaviors for ethanol. While both models of social interaction have been found to diminish drug reward-related behaviors, it is unclear whether there is any difference in effectiveness, particularly regarding ethanol drug reward. The goal of the present study was to determine whether social interaction, either through a social housing paradigm or a concurrent social reward-paired context, reduces reward-related behaviors for ethanol. While both models of social interaction have been found to diminish drug reward-related behaviors, it is unclear whether there is any difference in effectiveness, particularly regarding ethanol drug reward. The present study utilizes a conditioned place preference (CPP) paradigm to assess the conditioned ethanol approach behavior. The CPP paradigm has been used as a valid measure of drug reward-related behaviors [16]. Drug preference is established if, after the association between a drug and context is properly created, the animal spends significantly more time in the drug paired context. While this paradigm does not measure active drug-seeking behavior the way self-administration paradigms do, CPP allows for the assessment of the rewarding properties of a drug associated with a context, or drug associated preference behavior [17]. We hypothesized that rats who do not experience social interaction will show successful ethanol CPP while rats who experience social interaction, either through pair housing or socially-paired contextual conditioning, would attenuate ethanol CPP in rats.

## 2. Materials and Methods

### 2.1. Subjects

Adult male Sprague-Dawley rats (Charles River Laboratories, Houston, TX, USA, *N* = 26) were used in this study. Rats were single-housed or pair-housed on a reverse 12:12 light–dark cycle in a set temperature and humidity-controlled vivarium. During the experiment, animals were given chow and water ad libitum. All procedures were approved by the Institutional Animal Care and Use Committee (IACUC) of Trinity University. Experimental procedures, experimental animals, and sample sizes were conducted according to the ARRIVE guidelines [18].

### 2.2. Apparatus

The CPP apparatus (Panlab-Harvard Apparatus) was composed of two Plexiglass compartments (each: 30.0 cm length × 30.0 cm width × 34.0 cm height) that were connected by a central corridor (10.0 cm length × 8.0 cm width × 34.0 cm height). One compartment had a black floor and walls, while the other compartment had a white floor and walls. The central corridor had gray walls and a gray floor. The animal’s location and transitions between compartments were measured using pressure plates under the floors of the gray and black compartments and the data were relayed to the tracking software, PPCWIN, via a control panel (Panlab-Harvard Apparatus). The doors between the compartments were manually operated sliding doors. LED lighting was turned off during CPP sessions and a set of lights over the CPP chamber was set to the lowest setting throughout the paradigm.

### 2.3. Drugs

Koptec’s pure non-denatured ethanol (200 proof; King of Prussia, PA, USA) was diluted in 0.9% NaCl saline to a concentration of 20% *v*/*v* ethanol (1 g/kg; intragastric; i.g.).

### 2.4. Behavioral Protocol

#### 2.4.1. Housing

**Figure 1:**
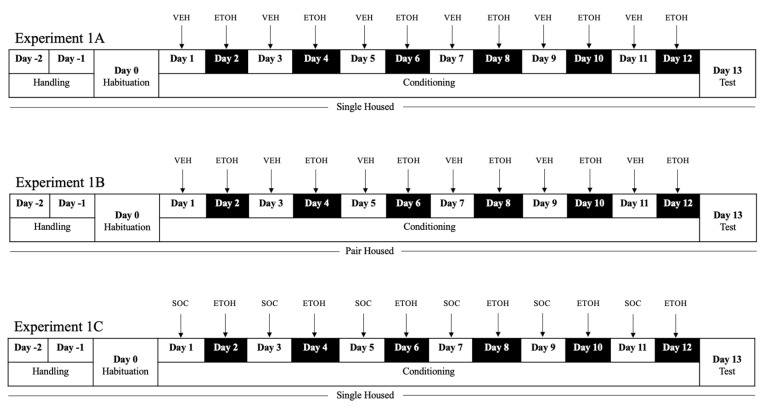
Experimental timeline and manipulation in experiment 1A, experiment 1B, experiment 1C. Ethanol-conditioning vs. saline or social-paired sessions were counterbalanced in all experiments for day and context. During conditioning, all rats received alternating conditioning trials in separate contexts. ETOH = ethanol; VEH = vehicle; SOC = social Interaction.

Two days prior to the habituation session, ethanol-naïve animals *(N* = 26) underwent handling to avoid anxious behavior throughout the paradigm. In experiment 1A (n = 10) and 1C, animals (n = 8) remained singly housed for the duration of the study. In experiment 1B (n = 8), animals were pair-housed. The conspecific roommates were matched by weight to avoid dominance in pairings. Pairings were checked daily for aggressive behavior that could obstruct the results. Animals were from the same litter and remained group housed until they were individually or pair-housed. The behavioral paradigm is outlined in Figure 1.

#### 2.4.2. Habituation

Behavioral training was run in the same 2-h period daily and began with a habituation trial to determine the baseline place preference. No ethanol or saline was administered. During habituation, all rats were individually placed in the gray central corridor. Doors were opened so the animal was allowed free access to both compartments for an entire 30-min session. During the session, the percentage of time spent within either compartment was calculated to determine the animal’s baseline preference. Whichever compartment the rat displayed less baseline preference for was designated as the ethanol-paired compartment during subsequent conditioning trials. Chambers were disinfected with 10% bleach and wiped down between rats throughout the paradigm to remove lingering scents that could influence preference or conditioning sessions.

#### 2.4.3. Conditioning

Rats were randomly assigned to the different treatment groups and counterbalanced so that all the rats did not start conditioning in the same compartment each day (i.e., subsequent sessions started in opposite chambers). Animals were also randomly assigned to ethanol drug groups. In experiments 1A and 1B, each group received six drug-paired sessions in the non-preferred compartment on alternating days (e.g., Days 1, 3, 5, 7, 9, 11) and six saline-paired sessions on the remaining days. In experiment 1C, each group received six drug-paired sessions in the non-preferred compartment on alternating days (e.g., days 1, 3, 5, 7, 9, 11) and six socially-paired sessions on the remaining days. Specifically, during socially-paired context conditioning, animals were placed in the saline paired context with another animal matched by weight. These pairings were consistent throughout the duration of the experiment. The socially-paired context served as a means of introducing acute social interaction to compete with the ethanol-paired reward. Animals received the vehicle or drug via intragastric administration five minutes prior to placement in the CPP chamber. Administration timepoints were chosen based on previous literature suggesting that ethanol administration 5 min prior to conditioning successfully established place preference [19]. Vehicle gavages were given to account for anxiety induced by the administration of ethanol gavages. Doors were closed during conditioning and rats were restricted to their specific context. Training sessions ran for 12 consecutive days and lasted 30-min each.

#### 2.4.4. Testing

On the thirteenth day (test day), animals underwent the same procedure as in habituation to determine whether ethanol CPP was established. No ethanol or saline was administered to the animals prior to testing.

### 2.5. Statistical Analysis

The amount of time and percentage of time spent in chambers were calculated using PPCWIN software. Percentage of time spent in the ethanol-paired chamber was calculated as time spent in drug-paired chamber/total time spent in all chambers. Time spent in the gray chamber was omitted as no time was spent in this chamber during the conditioning trials. A two-way ANOVA was conducted to identify differences in the percentage of time spent in the ethanol-paired chamber between the habituation and test in all three conditions (single-housed: ethanol vs. saline; pair-housed: ethanol vs. saline; single-housed: ethanol vs. conspecific). Post hoc Sidak’s multiple comparisons tests were used to determine differences in time spent in the ethanol-paired context between the habituation and test days in each condition group. A power analysis based on the data collected revealed an effect size of 1.058 and revealed that our sample size of n = 26 produced a statistical power of 0.99 (minimum n = 8 per condition). All data are presented as the mean ± S.E.M. and α was set at *p* < 0.05 with 95% confidence intervals.

## 3. Results

### Either Pair Housing and a Socially-Paired Context Diminished Ethanol Conditioned Place Preference

**Figure 2:**
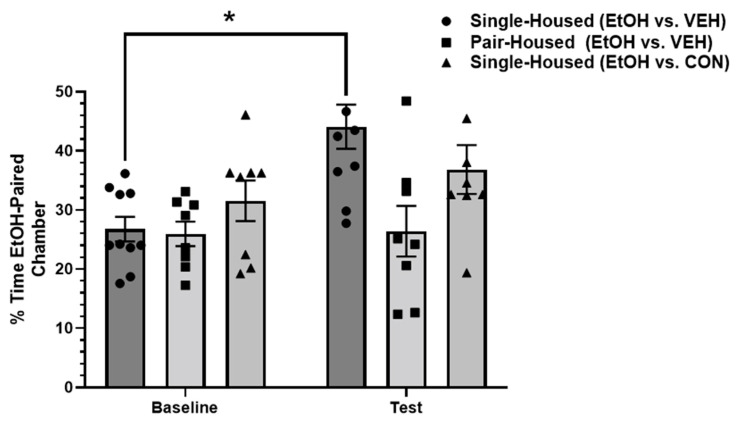
Percentage of time spent in the ethanol-paired chamber at the baseline and test. Single-housed animals that received ethanol or vehicle conditioning (EtOH vs. VEH) demonstrated an increased amount of time spent in the ethanol-paired context at test. Pair-housed animals that received ethanol or vehicle conditioning showed no increase in time spent in the ethanol-paired context at test. Single-housed animals that received ethanol or concurrent socially-paired context conditioning also showed no ethanol place preference. EtOH = ethanol-paired context; VEH = vehicle-paired context; CON = conspecific-paired context. * Denotes significant difference at *p* < 0.05.

A two-way ANOVA was conducted to examine the differences in the percentage of time spent in the ethanol-paired chamber across all three groups: single-housed (EtOH/VEH); pair-housed (EtOH/VEH); and single-housed (EtOH/Conspecific). A two-way ANOVA revealed a significant main effect of the treatment group [F(2,23) = 5.634, *p* < 0.05; η2 = 0.21] and a significant main effect of test [F(1,23) = 6.272, *p* < 0.05; η2 = 0.19]. A post hoc Sidak’s multiple comparisons test revealed a significant difference in percentage of time spent in the ethanol-paired chamber between the baseline (M = 26.75; SD = 6.57) and test (M = 44.1; SD = 11.84; *p* < 0.01) in the single-housed animals, suggesting the successful establishment of ethanol CPP. No significant difference was revealed in the time spent in the ethanol-paired chamber between the baseline (M = 25.95; SD = 5.85) and test (M = 26.4; SD = 12.1) in the pair-housed animals. Similarly, no significant difference was revealed in time spent in the ethanol-paired chamber between the baseline (M = 31.56; SD = 9.7) and test (36.85; SD = 11.71) in the single-housed animals that received concurrent conditioning in a socially-paired context. These results suggest that while single-housed animals successfully establish ethanol place preference, pair housing conditions or concurrent socially-paired context conditioning did not result in successful ethanol place preference (Figure 2).

## 4. Discussion

The present study revealed that both paired-housing conditions and a socially-paired context during conditioning effectively diminished ethanol CPP in male rats. Single-housed animals established ethanol CPP, as demonstrated by a significant increase in the percentage of time spent in the ethanol-paired chamber. However, when animals were pair-housed for the duration of the paradigm, this inhibited the ethanol CPP. Furthermore, single-housed animals, when concurrently conditioned to both an ethanol-paired context and a socially-paired context, displayed no significant increase in time spent in the ethanol-paired chamber. Overall, these findings suggest that social influence, either continual, through pair housing conditions, or acute, through a socially-paired context, impairs the acquisition of ethanol CPP.

Consistent with these results, ethanol CPP has been continually established in single-housed rodents [20,21]. Our findings are also consistent with several previous studies that have revealed an inhibitory effect of social interaction on drug-related behaviors. For example, social isolation increased morphine and amphetamine intake as well as cocaine-seeking behavior in rats [13,14,15,22,23,24]. For ethanol, social instability and isolation led to more robust ethanol CPP in rats [25] and increased ethanol intake [26,27]. However, the protective effects of pair housing on ethanol approach behaviors add to an already contradictory body of literature. In many studies, social interaction and social housing have increased drug-seeking behaviors and intake for morphine, cocaine, nicotine, and methamphetamine [6,13,28,29,30]. Specific to ethanol, social opportunity has been shown to increase ethanol consumption in rats [31,32,33]. Overall, the variable effects of social interaction on drug-seeking behavior may depend on drug dose, social rank of the animal, and the duration of their social interaction [34,35]. The anxiolytic effects of ethanol are also dependent on these factors [36].

In our study, we examined the specific effect of two forms of social interaction. In the continual social influence group, animals were pair-housed and allowed to maintain social contact throughout the duration of the experiment except for time spent in the conditioning chamber. In the acute social influence group, animals experienced a socially-paired context during conditioning and were exposed to social interaction during 30-min conditioning sessions on alternating days. The distinction between these two measures is important for understanding social interaction’s effect on ethanol approach behaviors, and how these behaviors translate to humans.

Although the specific mechanisms for social housing’s beneficial effects on CPP remain unanswered, it is possible that pair housing diminished social isolation-induced stress, which may in turn have affected ethanol CPP behavior. Studies have consistently found that social isolation increases stress- and anxiety-related behaviors, while social interaction conditions minimize the negative effects of stress [37]. This may be relevant, seeing as stress facilitates addiction related behaviors. For example, foot shock stressors were able to reinstate extinguished cocaine-, heroine-, and alcohol-seeking behaviors [38,39]. Furthermore, researchers have demonstrated that stress and anxiety enhance ethanol-seeking and consumption behavior. When rats were allowed intermittent social interaction, their ethanol intake was higher than those who were allowed continual social interaction [40]. Similarly, when animals who had constant access to social interaction were isolated for a period of time, their ethanol intake increased [27]. Therefore, social housing conditions may decrease ethanol CPP by decreasing the effects of stress on conditioned ethanol approach behavior relative to single-housed animals.

Alternatively, a socially-conditioned context may attenuate the effect of ethanol CPP through a competitive mechanism as opposed to an anxiolytic mechanism. Studies have shown that acute social interaction produces acute rewarding effects [9,41]. Furthermore, the rewarding effect of social interaction directly competes with drug-related rewards for several drugs of abuse such as amphetamine [15] and cocaine [14,23,24]. A study with concurrent social conditioning and cocaine conditioning found that social interaction CPP and cocaine CPP activated nearly the same brain regions to different extents [24]. Additionally, social interaction has been shown to reverse cocaine-induced translational changes in reward centers of the brain [14,23]. These findings are indicative of similar mechanisms underlying social CPP and drug CPP. A previous study found that individually housed rats were more sensitive to social reward [15]. Thus, the results of experiment 1C may be explained by a competition between social and ethanol reward, which present as reduced ethanol CPP.

Another possible mechanism for social interaction’s beneficial effect could be through facilitation of the endogenous oxytocin system. Social interaction, whether it be through paired-housing or socially-paired conditioning, may have diminished ethanol approach behavior through facilitation of the endogenous oxytocin system. Oxytocin is an endogenous neurochemical that the brain naturally releases in response to social interaction [42], maternal behavior [43], and in response to stress [41]. Oxytocin promotes anti-stress and restoration by reducing the blood pressure and cortisol levels and by mediating positive social interaction by decreasing anxiety [44]. It is hypothesized that the neurochemical oxytocin plays a predominant role in mediating the beneficial effects of social support on decreasing drug dependence. Studies have demonstrated that oxytocin effectively decreases the stress-induced reinstatement of ethanol reward behaviors. Additionally, oxytocin is implicated in the reward process, and consequently modulates the drug approach behavior. Oxytocin administration has been shown to attenuate cocaine [45], ethanol [46], and methamphetamine [47] drug-seeking behavior in rats. Oxytocin similarly protects against the rewarding effects of highly addictive substances such as sucrose [48]. Therefore, socially-paired conditioning and subsequent release of endogenous oxytocin may directly compete with drug approach behavior. This is supported by previous studies demonstrating that oxytocin within the VTA may facilitate the effects of social reward by acting on VTA dopaminergic projections to the nucleus accumbens [41]. Activation of these neurons facilitate social reward, but have no effect on cocaine CPP [41]. Taken together, it may be possible for oxytocin to underlie both the diminishing effect of social-housing and socially-paired conditioning on subsequent ethanol CPP expression.

The animal model used in the present study, CPP, is known to assess the rewarding property of substances and context-dependent reward memories [49]. The overall translational ability of these animal models has been debated. CPP allows for the assessment of drug reward without inducing tolerance or sensitization (Bardo & Bevins, 2000). However, the paradigm does not control for confounds such as novelty-seeking and inherent differences in animal preferences [17]. A recent meta-analysis of previous CPP studies validated the transferability of CPP paradigms to humans [50]. Therefore, the ability of both acute and continuous social interaction to attenuate ethanol-context approaching behavior in rats can be translated to humans. It is also important to note that previous studies have noted that specific behaviors (e.g., rearing, sniffing, proximal movements) may differ depending on the type of reinforcer used in the CPP paradigm [51], and may be used to determine the associative properties measured during CPP. The present study did not address this, although it is important for future studies to take this into consideration.

## 5. Conclusions

In conclusion, the present study demonstrates that social interaction reduces ethanol conditioned place preference and increases the understanding regarding the therapeutic effects of social support in alcohol addiction. Though the specific mechanisms have yet to be fully elucidated, this study builds on growing evidence suggesting that social interaction plays a key role in modulating addiction related behaviors. Furthermore, the conditions of social interaction, whether continuous through pair housing or acute through socially-paired contextual conditioning, is sufficient to decrease ethanol CPP. These results indicate that both social interaction conditions are capable of attenuating conditioned ethanol approach behavior. These results translate directly to human social interaction and highlight the importance of social support in addiction prevention and recovery programs for individuals suffering from AUD.

## Data Availability

The data presented in this study are available in the present article and on request from the corresponding author.
